# Feeding oxidized chicken byproduct meal impacts digestibility more than performance and oxidative status in nursery pigs

**DOI:** 10.1093/jas/skab029

**Published:** 2021-01-29

**Authors:** Carl A Frame, Elisabeth Huff-Lonergan, Brian J Kerr, Mariana Rossoni Serao

**Affiliations:** 1 Department of Animal Science, Iowa State University, Ames, IA, USA; 2 USDA-ARS National Laboratory for Agriculture and the Environment, Ames, IA, USA

**Keywords:** digestibility, growth, lipid oxidation, oxidative stress, protein oxidation

## Abstract

Rendered products from the meat industry provide quality proteins in diets for companion animals. These proteins are exposed to extreme temperatures during processing leading to the potential for decreased diet digestibility and subsequent growth performance. While this would impact production efficiency in livestock species, oxidized ingredients in companion animal diets may impact health and longevity. The objective of this study was to determine the extent to which a feedstuff containing oxidized protein and lipid affect diet digestibility, growth performance, and oxidative stress in nursery pigs. A total of 56 male pigs (21 d of age, initial body weight 5.51 ± 0.65 kg) were randomly assigned to one of the four dietary treatments in a 2 × 2 factorial arrangement with two levels of heat and two levels of antioxidant (AOX). Diets were fed for 35 d and growth performance was measured, while total tract digestibility and nitrogen (N) balance was determined during the trial on day 18–20. Blood plasma was collected on day 34 and jejunum, colon, and liver tissues were collected on day 35 to analyze for markers of oxidative stress. Average daily feed intake (ADFI) was reduced in pigs fed diets without AOXs (*P* = 0.02). Additionally, pigs consuming diets containing heated chicken byproduct (CBP) meal had decreased gain:feed (GF; *P* = 0.02). There was an interaction between heat and AOX (*P* = 0.02) where heating CBP reduced N digestibility in the presence of an AOX but did not have an impact when AOX was not present. The removal of AOX resulted in reduced GE digestibility (*P* < 0.01). Dry matter (*P <* 0.01), ash (*P <* 0.01), and protein (*P* < 0.01) digestibility were reduced (*P <* 0.01) as a result of heating. Furthermore, heating (*P =*0.01) as well as absence of AOX (*P =*0.01) resulted in reduced digestible energy. No difference was detected in N retention suggesting that oxidation reduces digestibility but has no impact on N utilization. This is supported by the fact that systemic oxidative stress was not consistently affected by heating or AOX inclusion. These results suggest that feeding pigs CBP containing oxidized proteins and lipids did not induce oxidative stress. However, feeding young pigs CBP containing oxidized proteins and lipids did result in reduced energy and nutrient digestibility as well as negatively affected feed efficiency. Because CBP is commonly used in companion animal diets, it is reasonable to revisit their impacts on those species.

## Introduction

Oxidative stress is believed to play a part in certain age-related diseases such as Alzheimer’s disease, Parkinson’s disease, inflammatory bowel disease, muscular dystrophy, and diabetes (Estévez and Luna, 2017). There is a growing concern of oxidative stress impacting companion animals as well. Oxidative stress is known to be a result of various environmental factors, but it may also be induced by the consumption of oxidized products (Estévez and Luna, 2017). It is hypothesized that consumption of severely oxidized products could further damage lipids, proteins, or DNA, resulting in an accumulation of reactive oxygen species (ROS).

When feed ingredients are exposed to heat, lipids and proteins within the ingredient are capable of forming ROS resulting in oxidation (Roldan et al., 2014; Feng et al., 2015; Zhang et al., 2017; Lindblom et al., 2018). These ROS can attack either proteins or lipids, leading to an increase in oxidation of both nutrients (Nielsen et al., 1985). During the rendering process, byproducts are exposed to high temperatures (i.e., >115 °C) to inactivate harmful pathogens and spoilage microbes (Anderson, 2006). Additionally, these products may be stored for extended periods of time allowing for further oxidation to occur. Therefore, rendered protein meals have the potential to contain oxidized lipids and proteins due to the production and handling.

Rendered by-products of the meat industry are commonly used in animal feeds and companion animal diets as a source of high-quality protein. Dry companion animal diets include protein at 31% (Hill et al., 2009), with some exceeding 50%, even when the minimum requirement for adult dogs and cats are 18% and 26%, respectively (AAFCO, 2019). These levels are much higher than in most production animal diets where feed costs drive formulation and protein is kept at a minimum. Among all rendered protein sources, poultry by-product meals are the most commonly used in premium pet foods due to their favorable amino acid and fatty acid profiles, and their positive impact on diet palatability (Aldrich, 2006). Considering the levels of protein in companion animal diets, which are often fed their entire life and their potential to contain oxidized products, these animals may be susceptible to oxidative stress, or other health/growth impacts.

It is unclear how dietary oxidized products impact companion animals. However, in livestock species, it has been shown that oxidative stress can reduce efficiency and increase production costs (Dibner et al., 1996; DeRouchey et al., 2004). Research in livestock has primarily focused on dietary lipid oxidation (Boler et al., 2012; Lindblom et al., 2017). However, a study where oxidized spray dried plasma was fed to pigs for 19 d, determined that oxidation reduced protein digestibility and increased crypt depth, but did not induce oxidative stress (Frame et al., 2020). Rendered proteins provide a valuable and realistic model to understand how dietary oxidized protein and lipid impact growth and health. Chicken by-product meal (CBP), which contains 70% protein and 13% lipid, is an ingredient that is widely used among the pet industry and has the potential to contain products of both protein and lipid oxidation. It is commonly recognized that lipid in this product can oxidize, and as such, manufacturers commonly utilize antioxidants (AOX) to limit oxidation (Aldrich, 2006). It could be hypothesized that increasing the degree of protein and lipid oxidation of CBP included in a diet would result in decreased digestibility of the diet as well as cause decreased performance and oxidative stress in pigs. Therefore, the objective of this study was to determine the extent to which oxidized protein and lipid will affect the digestibility of a diet, as well as the impact on performance and oxidative stress in pigs.

## Materials and Methods

The protocol for this experiment was reviewed and approved by The Institutional Animal Care and Use Committee at Iowa State University (Ames, IA).

### Animal management

A total of 56 male pigs at 21 d of age, 5.51 ± 0.65 kg, were individually housed in raised pens (43 cm × 116 cm) for 14 d followed by 7 d in metabolism pens (53 cm × 71 cm) to collect urine and feces, after which pigs were then moved back to individual pens for an additional 14 d. While in the individual pens, days 0–14 and 21–35, pigs were allowed ad libitum access to their respective diet. While housed in the metabolism pens, days 14–21, pigs were fed a defined amount of feed based on the first 14 d of feed intake to ensure complete consumption. During the entire study, pigs were allowed free movement and were allowed free access to water. Pigs remained on their respective diet for the entire 35 d trial.

### Diets and feeding

A basal diet utilizing CBP from a commercial source as the major protein source (23% inclusion) was formulated to meet NRC (2012) energy and nutrient requirements. Two diet phases were formulated to best match the nutrient requirement to the growth of the pig. Phase 1 was fed from days 0 to 20 including the metabolism collection period, while the phase 2 diet was fed from days 21 to 35. To achieve a 2 × 2 factorial arrangement of treatments, diets were created with CBP that did or did not include an AOX (mixed tocopherol) in combination with CBP being held at either 20 °C or being exposed to heat treatment (100 °C for 3 d). Vitamins and minerals were included in concentrations that met or exceeded the requirement for growing pigs NRC (2012), with no AOXs added to the final mixed diet, other than the mixed tocopherols added into the CBP. Of particular note, vitamin E was supplemented in the vitamin mix at 17.5 mg/kg diet to minimize vitamin levels above the NRC (2012) recommendations of 16 mg vitamin E/kg. Titanium dioxide was added as an indigestible marker to diets to allow for apparent total tract digestibility calculation. After mixing, feed was stored at 20 °C until fed.

### Sample collection

Pig and feeder weights were recorded at days 0 and 35 to determine growth performance data. During the metabolism trial, pigs were allowed 4 d of adaptation from days 14 to 17. Grab samples of feces and total urine was collected daily on days 18–20 and subsequently stored at −20 °C. Fecal samples were then dried at 100 °C and ground through a 1-mm screen prior to analysis. On days 34, 8 mL of blood was collected via jugular venipuncture, subsequently centrifuged at 1,000 × *g* for 10 min at 4 °C, with plasma collected and stored at −80 °C for further assays. On days 35, pigs were euthanized by captive bolt followed by exsanguination for harvesting of various tissues. Liver, jejunum, and colon were washed with phosphate-buffered saline (PBS), snap frozen in liquid nitrogen, and stored at −80 °C until further assayed. Additionally, jejunum samples were placed in 10% w/v formalin to be stained with hematoxylin and eosin (Iowa State University Veterinary Diagnostic Laboratory, Ames, IA). The resulting slides containing jejunum samples were analyzed for crypt depth and villi height (OLYMPUS BX 53/43 microscope with an attached DP80 Olympus camera, Olympus Waltham, MA). For each pig, 15 villus and crypt pairs with proper orientation were measured using computer software (OLYMPUS cellSens Dimension 1.16), averaged by pig, and reported as 1 value per pig.

### Chemical analysis

Ingredient, diets, and fecal samples were analyzed in duplicate for dry matter (DM; AOAC 930.01), ash (AOAC 942.05), N by using thermal combustion (AOAC 992.15), acid hydrolyzed ether extract (AOAC 2003.06), and gross energy (GE) using a bomb calorimeter (model 6200; Parr Instrument Co., Moline, IL) with benzoic acid (6,318 kcal GE/kg; Parr Instrument Co.) used for calibration. Urine was also analyzed for N using thermal combustion (AOAC 992.15). Titanium dioxide was determined according to the colorimetric method of Leone (1973). Complete diets and CBP were analyzed for carbonyls by solubilizing 1 g of sample in 50 mL of 5% w/v sodium dodecyl sulfate and incubating in a 50 °C water bath for 1 h (Soglia et al., 2016), followed by centrifugation for 10 min at 3,000 × *g* at 4 °C to remove any remaining insoluble particles with the supernatant used to analyze for carbonyls (Levine et al., 1990). Complete diets and CBP were also analyzed for TBARS as a measure of lipid oxidation (Buege and Aust, 1978).

### Oxidative stress assays

Tissue samples were analyzed for carbonyls (protein oxidation), malondialdehyde (MDA) by thiobarbituric acid reactive substances (TBARS; lipid oxidation), and 8-OH-2-deoxyguanosine (DNA damage). Plasma was tested for carbonyls and TBARS, and urine was analyzed for TBARS and 8-OH-2-deoxyguanosine. Tissues were prepared according to manufacturer’s instructions as previously described (Frame et al., 2020). Urine samples were diluted 1:750 prior to analyzing 8-OH-2-deoxyguanosine. Kits for TBARS, and 8-OH-2-deoxyguanosine kits were obtained from a commercial company (Cayman Chemical, Ann Arbor, MI) and performed according to manufacturer’s instructions. Carbonyls were analyzed as described previously (Levine et al., 1990; Frame et al., 2020).

### Calculations and statistical analysis

The following equations were used for calculating average daily feed intake (ADFI), average daily gain (ADG), and gain to feed (GF) using feeder and pig weights from days 0 and 35.


$$\begin{array}{ll} ADFI= & \;\;\frac{total\;feed\;intake,\;kg}{35\;days},\;ADG=\frac{total\;\;gain,\;kg\;}{35\;days},\;\\ & G:F=\frac{total\;gain,\;kg\;}{total\;feed\;intake,\;kg} \end{array}$$

After chemical analysis was completed on diets and fecal samples, digestibility was calculated using the following equation.


$$\begin{array}{l} \%\;\;Digestibility=100-\left[100\times \left(\frac{TiO_{2\left(diet\right)}}{TiO_{2\left(feces\right)}}\right)\times \left(\frac{Nutrient_{\left(feces\right)}}{Nutrient_{\left(diet\right)}}\right)\right]. \end{array}$$

Metabolizable energy (ME, in kcal/kg) was calculated by subtracting urine energy from the total digestible energy intake, then divided by total intake.


$$\begin{array}{l} ME=\frac{digestible\;energy\;\;intake,\;kcal-urinary\;energy,\;kcal}{Feed\;intake,\;kg}. \end{array}$$

Urinary energy values were calculated using the following equation based on the strong relationship between urinary nitrogen and urinary energy (Noblet and van Milgen, 2004).


$$\begin{array}{l} Urinary\;energy=192+31\times urinary\;nitrogen. \end{array}$$

A total of 56 pigs were randomly assigned to one of the four dietary treatment (*n* = 14 pigs per treatment). Pigs and treatments were evenly split into two separate rooms for the first 14 d and last 14 d of the study, using the room as a blocking factor. Data were analyzed as randomized complete block with treatments in a 2 × 2 factorial design with the main effects being AOX (with and without) and additional thermal processing (held at 20 °C, or heated at 100 °C for 3 d). During the study one pig died from disease unrelated to treatment. Therefore, samples were collected from 55 pigs resulting in 13 or 14 pig per treatment (*n* = 13 or 14). Initial body weight was used as covariates for analysis of all data. Results were analyzed by using the following model:


$$\begin{array}{l} \gamma_{ijk}=\mu +A_{i}+H_{j}+{\left(A\times H\right)}_{ij}+R_{ijk}+iBW_{ijk}+\varepsilon_{ijk}. \end{array}$$

In the above model, *γ* is the observed value, *μ* represents the overall mean, *A* is the *i*^th^ level for the fixed effect of AOX, *H* is the *j*^th^ level of the fixed effect of heat, *R*_*ijk*_ represents the room associated with *pig*_*ijk*_, *iBW*_*ijk*_ represents the initial body weight associated with *pig*_*ijk*_, and *ε*_*ijk*_ represents the random error associated with *γ*_*ijk*_ assuming that $∼N\left(0,I\sigma_{\varepsilon }^{2}\right)$. *P*-values ≤ 0.05 were considered significantly different, with *P*-value of 0.05 < *P* ≤ 0.10 considered trends. Data were analyzed using Proc GLM procedure of SAS (version 9.4; SAS) with means reported using LSMEANS using Tukey post hoc test. To assess how total consumption of oxidized products (total carbonyls or total TBARS) impacted growth and health parameters, irrespective of the factorial arrangement of treatments, regression coefficients were obtained by using the Proc Corr procedure of SAS (version 9.4; SAS). Total carbonyls or MDA consumed were calculated by multiplying total feed intake for each phase by carbonyls or MDA in the respective diet for each feeding phase then summed together.

## Results and Discussion

### Diets and composition

Carbonyl values for phase 1 were least for the diet that included an AOX and no heat, and greatest for the diet that did not include an AOX and was heated (24.7 vs. 29.4, respectively; Table 1). During phase 2, the inclusion of AOX resulted in lower carbonyl values in both the ingredient and diet, while heating CBP appeared to have a minimal impact on carbonyl values in the CBP or the final diet (Table 2). Additionally, TBARS was used to confirm lipid oxidation in both the CBP and the diets. With respect to lipid oxidation, TBARS was least for CBP with AOX and not as expected. However, TBARS were greatest in the CBP that was not heated and did not contain AOXs. This was the same for both feeding periods. While this was not expected, the final diets did not follow the same trend. The final diet with no heat and the inclusion of AOX was the least, while the diet with heat and without AOX was greatest for TBARS during phase 1 (2.62 vs. 3.63, respectively) and for phase 2 (2.27 vs. 3.33, respectively). This confirms that heat and no inclusion of AOX resulted in greater amount of oxidized protein and lipid in the final diet.

**Table 1.** Diet formulation and analyzed composition of diets during phase 1, as-fed basis^1^

	Treatment			
	AOX+ Heat−	AOX+ Heat+	AOX− Heat−	AOX− Heat+
Ingredient, %				
Corn	57.0	57.0	57.0	57.0
CBP + AOX^2^	23.15	–	–	–
Heated CBP + AOX	–	23.15		–
CBP^3^	–	–	23.15	–
Heated CBP	–	–	–	23.15
Plasma protein	5.0	5.0	5.0	5.0
Lactose	5.0	5.0	5.0	5.0
Whey protein	5.0	5.0	5.0	5.0
Soybean oil	2.65	2.65	2.65	2.65
Micro ingredints^4^	1.70	1.70	1.70	1.70
Titanium dioxide^5^	0.50	0.50	0.50	0.50
Analyzed composition				
DM, %	89.9	91.5	90.4	91.5
Ash, %	6.0	5.4	6.0	5.5
CP, %	25.8	24.6	25.9	26.1
GE, kcal/kg	4,794	4,716	4,762	4,678
Lipid, %	6.1	5.5	6.2	5.5
TBARS CBP, mg MDA/kg	4.75 ± 0.01	4.96 ± 0.05	9.67 ± 0.33	6.48 ± 0.33
Carbonyl CBP^6^, nmol/mg protein	24.4 ± 0.86	34.3 ± 1.67	44.9 ± 1.72	60.7 ± 1.34
TBARS final diet, mg MDA/kg	2.62 ± 0.19	3.24 ± 0.29	2.88 ± 0.23	3.63 ± 0.14
Carbonyl in final diet,^7^ nmol/mg protein	24.7 ± 0.19	26.9 ± 0.11	27.7 ± 0.38	29.4 ± 0.07

AOX, mixed tocopherol antioxidant; CBP, chicken by-product; DM, dry matter; CP, crude protein; GE, gross energy, TBARS, thiobarbituric acid reactive substances.

^1^Pigs were fed respective diets individually from days 0 to 20.

^2^AOX consisted of mixed tocopherols.

^3^CBP was either not heated or heated at 100 °C for 72 h.

^4^Micro-ingredients, as % of diet included 0.4% salt, 0.5% l-lysine, 0.17% dl-methionine, 0.16% l-threonine, 0.10% l-isoleucine, 0.09% l-valine, 0.06% l-tryptophan, 0.15% mineral premix (supplied per kg of complete diet: 9 ppm Cu, 120 ppm Fe, 120 ppm Zn, 7 ppm Mn, 0.2 ppm I, 0.2 ppm Se), 0.07% vitamin premix (supplied per kg of complete diet: 2,143 IU vitamin A, 245 IU vitamin D3, 17.5 IU vitamin E, 1.1 IU vitamin K, 3.9 mg riboflavin, 19.6 mg niacin, 9.5 mg pantothenic acid, and 18 μg vitamin B_12_).

^5^Titanium dioxide was used as an indigestible marker for determining digestibility.

^6^Protein carbonyls were analyzed as a measure of protein oxidation in CBP.

^7^Protein carbonyls were analyzed as a measure of protein oxidation in complete diet after mixing.

**Table 2.** Diet formulation and analyzed composition of diets during phase 2, as-fed basis^1^

	Treatment			
	AOX+ Heat−	AOX+ Heat+	AOX− Heat−	AOX− Heat+
% Diet				
Corn	64.74	64.74	64.74	64.74
CBP + AOX^2^	23.5	–	–	–
Heated CBP + AOX	–	23.5		–
CBP^3^	–	–	23.5	–
Heated CBP	–	–	–	23.5
Plasma protein	2.5	2.5	2.5	2.5
Lactose	2.5	2.5	2.5	2.5
Whey protein	2.5	2.5	2.5	2.5
Soybean oil	2.18	2.18	2.18	2.18
Micro ingredints^4^	1.58	1.58	1.58	1.58
Titanium dioxide^5^	0.50	0.50	0.50	0.50
Analyzed composition				
DM, %	90.2	90.1	89.8	90.8
Ash, %	5.0	4.9	4.9	5.0
CP, %	23.6	24.0	23.5	24.8
GE, kcal/kg	4,669	4,767	4,645	4,743
Lipid, %	7.8	7.8	7.2	8.3
TBARS CBP, mg MDA/kg	4.71 ± 0.03	4.95 ± 0.08	9.76 ± 0.27	5.45 ± 0.13
Carbonyl CBP^6^, nmol/mg protein	30.3 ± 1.28	31.8 ± 0.42	61.6 ± 3.21	62.8 ± 0.92
TBARS final diet, mg MDA/kg	2.27 ± 0.01	2.68 ± 0.18	3.24 ± 0.17	3.33 ± 0.23
Carbonyl in final diet,^7^ nmol/mg protein	21.6 ± 0.05	21.4 ± 0.27	26.7 ± 0.03	26.7 ± 0.09

AOX, mixed tocopherol antioxidant; CBP, chicken byproduct; DM, dry matter; CP, crude protein; GE, gross energy; TBARS, thiobarbituric acid reactive substances.

^1^Pigs were fed respective diets individually from days 21 to 25.

^2^AOX consisted of mixed tocopherols.

^3^CBP was either not heated or heated at 100 °C for 72 h.

^4^Micro-ingredients, as % of diet included 0.4% salt, 0.47% l-lysine, 0.13% dl-methionine, 0.15% l-threonine, 0.07% l-isoleucine, 0.08% l-valine, 0.06% l-tryptophan, 0.15% mineral premix (supplied per kg of complete diet: 9 ppm Cu, 120 ppm Fe, 120 ppm Zn, 7 ppm Mn, 0.2 ppm I, 0.2 ppm Se), 0.07% vitamin premix (supplied per kg of complete diet: 2,143 IU vitamin A, 245 IU vitamin D3, 17.5 IU vitamin E, 1.1 IU vitamin K, 3.9 mg riboflavin, 19.6 mg niacin, 9.5 mg pantothenic acid, and 18 μg vitamin B_12_).

^5^Titanium dioxide was used as an indigestible marker for determining digestibility.

^6^Protein carbonyls were analyzed as a measure of protein oxidation in CBP.

^7^Protein carbonyls were analyzed as a measure of protein oxidation in complete diet after mixing.

### Digestibility

Results for apparent total tract digestibility (ATTD) of DM, ash, crude protein (CP), GE, and lipid are summarized in Table 3. A heat × AOX interaction was observed on ATTD of N (*P* = 0.02), where CBP containing AOX resulted in increased ATTD of N when CBP was not heated (79.3% vs. 75.2%, AOX added vs. no added AOX, respectively), but had no effect when CBP was heated (70.9% vs. 72.0%, added AOX vs. no added AOC, respectively). Heating resulted in decreased DM, ash, CP, and GE digestibility (*P <* 0.01). These result are consistent with Chen et al. (2015) which concluded that in growing broilers, as feed became more oxidized, DM and CP digestibility decreased. Furthermore, Frame et al. (2020) reported that that DM and CP was less digestible in diets containing oxidized spray dried plasma fed to nursery pigs. Heat-induced oxidation has been shown to alter the structure of proteins resulting in aggregation (Promeyrat et al., 2010) where it has been proposed that oxidation of proteins decreases their susceptibility to proteolysis resulting in decreased digestibility (Sante-Lhoutellier et al., 2007; Wu et al., 2014). Others have shown that in pigs fed oxidized protein, a decrease in digestibility of proteins and DM was a result of decreased trypsin and amylase activity (Zhang et al., 2016) in addition to decreased lipase and protease activity (Chen et al., 2015).

**Table 3.** Digestibility and N balance of diets containing varying levels of dietary oxidized protein fed to growing pigs

	Treatment^1^					*P*-value		
	AOX+ Heat−	AOX+ Heat+	AOX− Heat−	AOX− Heat+	SEM	AOX	Heat	AOX × Heat
Digestibility								
DM, %	91.1	89.4	90.7	88.6	0.49	0.24	<0.01	0.67
Ash, %	70.4	65.9	68.4	64.7	0.79	0.05	<0.01	0.62
N, %	79.3^a^	70.9^c^	75.2^b^	72.0^c^	1.07	0.17	<0.01	0.02
GE, %	86.7	82.9	84.4	82.5	0.65	0.05	<0.01	0.14
Lipid, %	77.0	74.7	77.0	73.6	1.63	0.88	0.12	0.58
Energy and N balance								
DE, kcal/kg	4,158	3,908	4,019	3,861	31	0.01	<0.01	0.15
ME, kcal/kg	4,049	3,795	3,912	3,755	33	0.01	<0.01	0.15
N Retained^2^, %	82.4	79.0	80.5	80.4	1.4	0.85	0.26	0.26

DM, dry matter; AOX, antioxidant; N, nitrogen; GE, gross energy; DE, digestible energy; ME, metabolizable energy.

^a–c^Difference in superscript indicate significant interaction, *P* < 0.05.

^1^Difference in dietary treatments were achieved by the inclusion or absence of AOX in CBP, and with or without additional heat treatment (100 °C for 72 h) of CBP. There were 14 individually penned gilts per treatment with an initial BW of 5.51 ± 0.65 kg. Pigs were fed for 14 d, after which they were placed in metabolism crates for 4 d followed by a 3-d collection.

^2^N retention was calculated as a percentage of N retained over N absorbed.

Previously it has been suggested that oxidation may not result in differences in digestible energy (DE), but may lead to differences in ME (Frame et al., 2020). In the current study, increased oxidation as a result of no AOX inclusion resulted in decreased DE (*P =* 0.01) and ME (*P <* 0.01). Likewise, there was decreased DE (*P =* 0.01) and ME (*P <* 0.01) of heated diets. Similar to this study, Rosero et al. (2015) also found a decrease in DE in pigs fed diets containing oxidized lipids.

Nitrogen balance was also examined to determine if absorbed N (i.e., amino acids) were damaged by oxidation and were unusable to the body. Result showed no difference in N balance across all treatments (Table 3). This suggests that differences in ME across treatments was driven by diet digestibility, rather than differences in metabolism.

### Growth performance

While it is unlikely that livestock would be consuming rendered meals at inclusion levels used in this study due to cost, it is important to understand how feeding an oxidized ingredient can impact growth performance. During the 34 d trial, no interaction was observed between AOX and heat for ADG, ADFI, or GF (Table 4). No difference was observed for ADG due to heat or AOX inclusion (*P* > 0.10). Pigs consuming diets with no AOX inclusion had decreased ADFI (*P* = 0.02). Previous studies have also shown a decrease in ADFI with greater concentrations of oxidized lipids fed to pigs (DeRouchey et al., 2004; Liu et al., 2014; Rosero et al., 2015). In the present study, CBP contained 13.4% lipid and was the only source of oxidized lipid in the diet. Therefore, given the inclusion rate of CBP of 23.15% and 23.50% for phases 1 and 2, the final diet contained 3.10% and 3.15% of oxidized lipid for phases 1 and 2, respectively. The inclusion level of oxidized lipid in this study is less than the 6% inclusion (DeRouchey et al., 2004; Rosero et al., 2015) or 10% inclusion (Liu et al., 2014) used in other studies. However, these other studies were solely focused on lipid oxidation whereas this study examined the impact of both oxidized lipids and proteins. With respect to GF, pigs consuming diets containing heated CBP had decreased GF (*P* = 0.02). Studies examining lipid oxidation have also observed this decrease of GF in broilers (Dibner et al., 1996; Takahashi and Akiba, 1999; Tavarez et al., 2011; Hung et al., 2017). With respect to dietary protein oxidation, when broilers were fed oxidized soy protein isolate (Wu et al., 2014; Chen et al., 2015) or when pigs were fed oxidized spray dried plasma (Frame et al., 2020), no difference in GF was observed. Therefore, the decrease in GF observed in this study could be a result of either lipid oxidation products or due to consumption of oxidized protein, which are supported with the decreased digestibility of DM, ash, CP, and GE.

**Table 4.** Growth performance data for pigs fed diets containing varying levels of dietary oxidized protein

	Treatment^1^					*P*-value		
Item	AOX+ Heat−	AOX+ Heat+	AOX− Heat−	AOX− Heat+	SEM	AOX	Heat	AOX × Heat
Growth performance								
Initial BW, kg	5.85	5.41	5.43	5.38	0.172	0.20	0.28	0.16
Final BW, kg	17.53	17.06	16.71	15.62	0.693	0.11	0.27	0.66
ADG, kg	0.33	0.34	0.32	0.29	0.016	0.11	0.57	0.27
ADFI, kg	0.49	0.52	0.46	0.47	0.017	0.02	0.48	0.53
GF	0.68	0.66	0.69	0.63	0.016	0.47	0.02	0.22

ADG, average daily gain; ADFI, average daily intake; GF, gain to feed ratio; AOX, antioxidant; CBP, chicken byproduct.

^1^Difference in dietary treatments were achieved by the inclusion or absence of AOX in CBP, and with or without additional heat treatment (100 °C for 72 h) of CBP. There were 14 individually penned gilts per treatment with an initial BW of 5.51 ± 0.65 kg. Pigs were fed for 35 d. Initial body weight was used as covariates for analysis of all data.

### Gut morphology

Histology samples from the jejunum were analyzed for villus height, crypt depth, and villus to crypt ratio (Table 5). A significant heat × AOX interaction was observed where the absence of AOX resulted in an increase in crypt depth in pigs fed CBP that were not heated (224 vs. 245 μm, respectively), but AOX had no effect when CBP was heated (237 vs. 232 μm, respectively, *P* = 0.03). Pigs consuming diets with no AOX inclusion had increased villus height compared with pigs consuming diets with added AOX (674 vs. 576 μm, respectively; *P <* 0.01). This difference was also reflected in the villi to crypt ratio (VC) where pigs fed diet without AOX had increased VC compared with diets containing AOX (3.20 vs. 2.83, respectively; *P <* 0.01). These results are consistent with Rosero et al. (2015) who reported increased villus height in pigs consuming oxidized soybean oil for 35 d where they proposed that lipid oxidation stimulated enterocyte proliferation as a result of oxidative stress in the small intestine, thus leading to changes in morphology. Therefore, consumption of oxidized products may increase crypt depth or villus height. We speculate that this increase in villus height may be a response to the decrease in apparent total tract digestibility in order to capture more nutrients.

**Table 5.** Histology data for pigs fed diets containing varying levels of dietary oxidized protein

	Treatment^1^					*P*-value		
Item	AOX+ Heat−	AOX+ Heat+	AOX− Heat−	AOX− Heat+	SEM	AOX	Heat	AOX × Heat
Histology^2^								
Villi height, μm	577	574	688	660	11.4	<0.01	0.19	0.27
Crypt depth, μm	224^b^	237^ab^	245^a^	232^ab^	5.9	0.19	0.97	0.03
Villi:crypt ratio	2.92	2.74	3.16	3.23	0.09	<0.01	0.53	0.14

AOX, antioxidant; CBP, chicken by-product.

^a,b^Difference in superscript indicate significant interaction.

^1^Difference in dietary treatments were achieved by the inclusion or absence of AOX in CBP, and with or without additional heat treatment (100 °C for 72 h) of CBP. There were 14 individually penned gilts per treatment with an initial BW of 5.51 ± 0.65 kg. Pigs were fed for 35 d.

^2^Jejunum samples were stained with hematoxylin and eosin and analyzed for crypt depth and villi height where 15 villus and crypt pairs with proper orientation were averaged by pig and reported as 1 value per pig.

### Oxidative stress

It should be noted that in general the carbonyl values in tissues and plasma observed in the current study were greater than other studies examining dietary oxidized protein (Zhang et al., 2017; Frame et al., 2020). This is likely due to the severity of oxidation in CBP (24.4–62.8 nmol carbonyl/mg protein) observed in the current study compared with other studies examining spray dried plasma (2.7–8.1 nmol carbonyl/mg protein, Frame et al., 2020) or soy protein isolate (7.1–10.4 nmol carbonyl/mg protein, Zhang et al., 2017).

Results for markers of oxidative stress measured in tissues and plasma are reported in Table 6. In plasma, no differences were observed on carbonyls. An unexpected difference was observed where pigs consuming a diet with no added AOX exhibited less lipid damage in the plasma compared to diets containing AOX as measured by TBARS (7.25 vs. 9.98 μM MDA, respectively; *P <* 0.01). In the jejunum, no significant interaction between heat and AOX was observed for protein, lipid, or DNA damage. A tendency was observed for protein damage where pigs consuming diets containing heated CBP tended to have less carbonyl formation compared with pigs fed unheated CBP (338 vs. 406 nmol/mg protein, respectively; *P =* 0.10). No difference was observed in lipid and DNA damage (8-OH-2-deoxyguanosine). As the main site of absorption, it would be expected that tissue from the small intestine would have the greatest exposure to dietary oxidized product, resulting in the most damage. However, these results would suggest that the jejunum was not in an oxidative stress status.

**Table 6.** Measures of oxidative stress for pigs fed diets containing varying levels of dietary oxidized protein

	Treatment^1^					*P*-value		
Measure of oxidation	AOX+ Heat−	AOX+ Heat+	AOX− Heat−	AOX− Heat+	SEM	AOX	Heat	AOX × Heat
Plasma								
Carbonyl, nmol/mg protein	67.2	52.8	59.7	52.6	7.3	0.60	0.15	0.62
TBARS, μM MDA	9.85	10.11	6.54	7.95	0.74	< 0.01	0.27	0.45
Jejunum								
Carbonyl, nmol/mg protein,	410	361	401	314	40	0.50	0.10	0.64
TBARS, μM MDA	4.41	3.85	3.68	4.40	0.45	0.85	0.86	0.17
8-OH-2-deoxyguanasine, pg/ml	120	74	110	113	21	0.48	0.31	0.25
Colon								
Carbonyl, nmol/mg protein	79.0	80.1	89.7	89.8	14.1	0.47	0.97	0.97
TBARS, μM MDA	12.56^ab^	6.49^b^	8.95^ab^	14.88^a^	2.15	0.24	0.97	< 0.01
8-OH-2-deoxyguanasine, pg/ml	140	171	174	171	32	0.60	0.66	0.59
Liver								
Carbonyl, nmol/mg protein	443	439	572	293	69	0.91	0.05	0.06
TBARS, μM MDA	5.39	5.36	6.09	5.19	0.32	0.43	0.16	0.19
8-OH-2-deoxyguanasine, pg/ml	352	283	337	348	67	0.71	0.66	0.56
Urine								
TBARS, μM MDA	7.67	8.61	9.68	10.02	1.04	0.10	0.54	0.78
8-OH-2-deoxyguanasine, ng/ml	363	329	309	346	29	0.54	0.95	0.24

AOX, antioxidant; TBARS, thiobarbituric acid reactive substances.

^a,b^Difference in superscript indicate significant interaction.

^1^Difference in dietary treatments were achieved by the inclusion or absence of AOX in CBP, and with or without additional heat treatment (100 °C for 72 h) of CBP. There were 14 individually penned gilts per treatment with an initial BW of 5.51 ± 0.65 kg fed for 35 d. Blood plasma was collected on day 34 and tissue samples were collected on day 35.

Given the observed decrease in digestibility, it might be speculated that oxidized products pass the small intestine and end up in the hind gut. However, no difference for colonic protein or DNA damage was detected. A significant heat × AOX interaction on colon TBARS was observed where pigs fed diets containing heated CPB, the absence of AOX increased lipid damage (14.88 vs. 6.49 μm MDA; *P* < 0.01). However, as no difference was observed for protein or DNA damage in the colon, it appears that colonic tissue was not critically affected by heating CBP or the addition of AOX.

A significant difference was observed for liver carbonyls, where pigs fed diets containing heated CBP had decreased protein damage compared to pigs fed unheated CBP (360 vs. 508 nmol/mg protein, respectively; *P* = 0.05). This unexpected outcome was similar to observations in both the plasma and jejunum tissue. There was no interaction between heat and AOX, or main effect of heat or AOX on liver TBARS or 8-OH-dG (*P* > 0.10).

Urine was analyzed for markers of DNA or lipid damage because these products are specifically concentrated in the kidney and excreted in the urine (Wu et al., 2004; Mateos and Bravo, 2007). Proteins are not typically excreted in the urine and thus, carbonyls were not evaluated (Lindblom et al., 2018). There was no interaction between heat or AOX for either urinary TBARS or 8-OH-dG, and there was no main effect of heat or AOX for urinary 8-OH-dG excretion (*P* > 0.10). There was a trend observed for urinary TBARS where urine from pigs fed diets containing heated CBP tended to have greater lipid damage than pigs fed unheated CPB (9.31 vs. 8.66 μM MDA, respectively; *P =* 0.10).

Of all measures of lipid, protein, and DNA damage, TBARS in the colon and urine was the only measure to increase as a result of increase oxidation. In contrast to expectations, TBARS in the plasma and carbonyls in the jejunum and liver decreased as a result of increased oxidation. This unexpected outcome may be a result of the body responding by clearing damaged lipids and protein, thus leading to lipid oxidation products decreasing in tissues, while increasing in the urine and colon.

### Regression analysis

Regressions were conducted irrespective of the factorial arrangement of treatments to relate total consumption of oxidized products to growth and health parameters (Table 7). During the first feeding phase of the study (days 0–21), only N retention was negatively correlated with total carbonyls consumption (*P <* 0.01), with no other digestibility or retention values being correlated to total carbonyls. Relative to TBARS correlation to digestibility or retention values during the first feeding phase, GE digestibility (*P <* 0.01), dietary ME (*P =* 0.05), and N retention (*P <* 0.01) were negatively correlated with total MDA (TBARS) consumption. Contrary to the previous factorial analysis where N retention was not impacted by treatment, N retention was negatively related with increased oxidation of proteins and lipids suggesting a difference in protein metabolism. This relationship may be impacted by damaged amino acids being absorbed and metabolized and excreted. This is the first of any study to observe such relation.

**Table 7.** Pearson correlation coefficients between total protein carbonyls or MDA consumed and performance and oxidative stress parameters in pigs fed diets containing varying levels of dietary oxidized lipid and protein^1^

Parameter	Total carbonyls consumed,^2^ days 0–20	Total MDA consumed, days 0–20^2^	Total carbonyls consumed,^3^ days 0–35	Total MDA consumed, days 0–35^3^
GE digestibility	–	−0.53 <0.01	–	–
ME	–	−0.26 0.05	–	–
N retention	−0.39 <0.01	−0.36 <0.01	–	–
ADG	–	–	0.69 <0.01	0.54 <0.01
ADFI	–	–	0.84 <0.01	0.69 <0.01
Villus height	–	–	0.40 <0.01	0.41 <0.01
Urinary TBARS	–	–	−0.40 <0.01	−0.27 0.05

GE, gross energy; ME, metabolizable energy; N, nitrogen; ADG, average daily gain; ADFI, average daily feed intake; TBARS, thiobarbituric acid reactive substances.

^a,b^Difference in superscript indicate significant interaction.

^1^Difference in dietary treatments were achieved by the inclusion or absence of AOX in CBP, and with or without additional heat treatment (100 °C for 72 h) of CBP. There were 14 individually penned gilts per treatment with an initial BW of 5.51 ± 0.65 kg fed for 35 d.

^2^Total carbonyl or MDA consumed was calculated by multiplying the total feed intake from days 0 to 20 by the carbonyl or MDA content in their respective diets.

^3^Total carbonyl or MDA consumed was calculated by multiplying the total feed intake from days 0 to 35 by the carbonyl or MDA content in their respective diets.

Over the entire study, parameters that were positively correlated with total carbonyls consumed were ADG (*P <* 0.01), ADFI (*P <* 0.01), and villus height (*P <* 0.01), while urinary TBARS were negatively correlated (*P <* 0.01) to total carbonyl consumption. Similarly, parameters that were positively correlated with total MDA (TBARS) consumed over the entire study were ADG (*P <* 0.01), ADFI (*P <* 0.01), and villus height (*P <* 0.01), while urinary TBARS was negatively correlated (*P <* 0.01). The relation between dietary oxidized products and villus height further support that dietary oxidized products results in an increased surface area of the jejunum. As with any correlation, further investigation needs to be conducted as to determine the cause of these relationships.

In plasma, jejunum, and liver tissues, pigs fed diets with greater carbonyl and TBARS values had decreased lipid and protein damage. This is an unexpected outcome and no other studies have shown a decrease in these markers with feeding a more oxidized diet. The unexpected decrease in protein and lipid damage in pigs consuming oxidized CBP could be a result of adaptation. It is possible that during the 35 d period, pigs fed oxidized CBP were able to upregulate AOX machinery. This would allow for clearing of oxidized products at the level of tissue and lead to excreting oxidized products in the urine or feces. These possibilities warrant more investigation as to whether end products can induce oxidative stress.

While significant differences were observed in select tissues, consistent damage of protein, lipids, and DNA across multiple tissues was not observed. Therefore, results from this study indicate that the levels of dietary oxidized protein and lipid fed herein do not induce systemic oxidative stress. However, oxidation did decrease DM, ash, and CP digestibility which supports the decrease in GF in pigs consuming oxidized CBP. In addition, villus height increased with increased inclusion of oxidized protein in the diet suggesting the pig may be trying to increase their absorptive surface area, which are consistent with previous work (Rosero et al., 2015).

While quantifiable data was not able to be obtained, an anecdotal difference was observed in hair and skin quality. Pigs fed oxidized CBP appeared to have thicker, rough hair with more dandruff, while pigs fed less oxidized CBP appeared to have a normal hair and skin quality. Considering sulfur containing amino acids are the most susceptible to oxidation, and these amino acids are vital in hair formation, future work should consider possible measures of hair quality. It is proposed that as a result of oxidation, damage to susceptible amino acids results in deficiencies in these components. This is especially important when considering companion animals, as coat and hair quality is a defining factor of acceptable companion animal diets.

Although livestock diets would not include rendered meals at the levels fed in the current study, the main concern of oxidation in ingredients appears to be the digestibility of energy and nutrients. In contrast, companion animals are fed higher levels of animal protein-by- products for the duration of the animal’s lifespan. Thus, the long-term effects of consuming oxidized proteins and lipid products should be further investigated. Additionally, the potential for oxidized ingredient to reduce diet digestibility should be considered in formulating companion animal diets.

## Abbreviations

ADFI: average daily feed intake
ADG: average daily gain
AOX: antioxidant
ATTD: apparent total tract digestibility
BW: body weight
CBP: chicken byproduct meal
CP: crude protein
DE: digestible energy
GE: gross energy
GF: gain to feed ratio
MDA: malondialdehyde
ME: metabolizable energy
N: nitrogen
PBS: phosphate buffered saline
ROS: reactive oxygen species
TBARS: thiobarbituric acid reactive substances
VC: villi to crypt ratio
